# The Ruler Protein EscP of the Enteropathogenic *Escherichia coli* Type III Secretion System Is Involved in Calcium Sensing and Secretion Hierarchy Regulation by Interacting with the Gatekeeper Protein SepL

**DOI:** 10.1128/mBio.01733-16

**Published:** 2017-01-03

**Authors:** Lihi Shaulov, Jenia Gershberg, Wanyin Deng, B. Brett Finlay, Neta Sal-Man

**Affiliations:** a The Shraga Segal Department of Microbiology, Immunology and Genetics, Faculty of Health Sciences, Ben-Gurion University of the Negev, Beer-Sheva, Israel; b Michael Smith Laboratories, University of British Columbia, Vancouver, British Columbia, Canada; Yale University School of Medicine; University of Mississippi Medical Center

## Abstract

The type III secretion system (T3SS) is a multiprotein complex that plays a central role in the virulence of many Gram-negative bacterial pathogens. To ensure that effector proteins are efficiently translocated into the host cell, bacteria must be able to sense their contact with the host cell. In this study, we found that EscP, which was previously shown to function as the ruler protein of the enteropathogenic *Escherichia coli* T3SS, is also involved in the switch from the secretion of translocator proteins to the secretion of effector proteins. In addition, we demonstrated that EscP can interact with the gatekeeper protein SepL and that the EscP-SepL complex dissociates upon a calcium concentration drop. We suggest a model in which bacterial contact with the host cell is accompanied by a drop in the calcium concentration that causes SepL-EscP complex dissociation and triggers the secretion of effector proteins.

## IMPORTANCE

The emergence of multidrug-resistant bacterial strains, especially those of pathogenic bacteria, has serious medical and clinical implications. At the same time, the development and approval of new antibiotics have been limited for years. Recently, antivirulence drugs have received considerable attention as a novel antibiotic strategy that specifically targets bacterial virulence rather than growth, an approach that applies milder evolutionary pressure on the bacteria to develop resistance. A highly attractive target for the development of antivirulence compounds is the type III secretion system, a specialized secretory system possessed by many Gram-negative bacterial pathogens for injecting virulence factors (effectors) into host cells. In this study, we shed light on the molecular mechanism that allows bacteria to sense their contact with the host cell and to respond with the timed secretion of effector proteins. Understanding this critical step for bacterial virulence may provide a new therapeutic strategy.

## INTRODUCTION

Gram-negative bacterial pathogens, such as pathogenic *Escherichia coli* and *Salmonella*, *Shigella*, *Yersinia*, and *Pseudomonas* spp., are causative agents of serious human diseases ranging from lethal diarrhea to the plague that account for millions of deaths annually worldwide. These pathogens all utilize a common syringe-like protein complex, termed the type III secretion system (T3SS) or injectisome, which injects virulence factors from the bacterial cytoplasm directly into the host cell (1, 2). This process is essential for the virulence of these bacterial pathogens, since the injected proteins—termed effectors—manipulate key host cell pathways (e.g., cell cycle, immune responses, cytoskeletal organization, metabolic processes, and intracellular trafficking) that ultimately promote bacterial replication, disease, and transmission (1, 3–5).

The T3SS is a syringe-like structure that comprises about 20 different proteins. This large protein complex consists of three major substructures: a basal body embedded within the bacterial membranes, an extracellular needle that bridges the extracellular space between the bacteria and the host cell, and a pore-forming complex, termed the translocon, which forms a channel in the membrane of the host cell. To ensure that translocation of the effector proteins into the host cells is efficient and timely, the secretion process is tightly regulated and consists of three groups of secreted substrates (summarized in reference 6). The first group of substrates that travels through the secretion apparatus (“early” substrates) comprises the inner rod and the extracellular needle proteins, both of which are structural proteins that assemble to form substructures within the T3SS. Upon completion of the needle structure, components of the translocon and the filament (a polymer of the EspA protein, uniquely present in the T3SS of *E. coli*) are secreted (translocators; “intermediate” substrates). Finally, once the connection of the T3SS to the host cell is complete, the secretion of the effector proteins (“late” substrates) is initiated. It was previously suggested that two substrate specificity switching events are at the heart of the regulation of this hierarchical secretion. Two principal models were proposed for the extensively studied first switch, between early and intermediate substrates, which was suggested to involve (i) a ruler protein that measures the length of the needle (7–12) and that switches substrate specificity, presumably via its interaction with an integral membrane protein from the EscU/FlhB/SpaS/Spa40/YscU family (8, 13–20). Members of this family of proteins undergo an autocatalytic cleavage event that allows them to alter their conformations and to switch substrate secretion specificity. Alternatively, the switch in specificity may entail (ii) completion of the inner rod substructure, which triggers conformational changes on the cytoplasmic side of the injectisome (21, 22).

The second substrate specificity switch, from intermediate to late substrates, was assumed to start only upon the completion of translocon assembly and the formation of a continuous conduit connecting the bacterial cytosol to the host cytosol. Although far from being understood, the switching event was suggested to involve a protein that binds to the T3SS and functions as a gatekeeper to selectively inhibit the secretion of effectors until it dissociates from the T3SS (23–26). On the basis of evidence that the translocator and effector secretion events are triggered by different environmental factors (e.g., serum albumin, bile salts, low calcium levels, and Congo red) (27–32), it was suggested that the dissociation of the gatekeeper protein occurs in response to an environmental factor. However, the nature of this signal and the molecular mechanism of how it is sensed by the bacteria were so far unknown.

In enteropathogenic *E. coli* (EPEC), which is a major cause of pediatric diarrhea (33), two cytosolic proteins, SepL and SepD, were suggested to be involved in regulation of the substrate secretion switch between translocators and effectors (34, 35). SepL is a known member of the gatekeeper family of proteins, which includes YopN in *Yersinia*, InvE and SsaL in *Salmonella*, and MxiC in *Shigella* (7). SepD, on the other hand, is not well conserved in other pathogens, and as such, it has a single, functional homolog, SpiC, which was identified in *Salmonella* pathogenicity island 2 (36–38). In addition, the YscB protein was suggested to belong to the SepD family of proteins because of its analogous position within the *Yersinia* pathogenicity island (39). Deletion of either *sepL* or *sepD* abrogated the secretion of translocators and resulted in the hypersecretion of effectors (34). Moreover, *sepL* and *sepD* null strains of the related murine pathogen *Citrobacter rodentium* were avirulent (40). In addition, in a mechanism similar to that observed in *Yersinia* spp. (28, 32), the extracellular calcium concentration was found to regulate the switch between translocators and effectors in EPEC (34, 41). Collectively, these data indicate that SepL and SepD are involved in the switch between the secretion of intermediate and late substrates in EPEC.

In this study, we found that the EPEC protein EscP, which was previously suggested to function as the ruler protein and is involved in the first substrate specificity switch (42), is also involved in regulation of the second substrate specificity switch. We showed that the EPEC Δ*escP* mutant is calcium blind and its type III secretion (T3S) protein profile exactly resembles that of wild-type (WT) EPEC grown in Ca^2+^-free medium. Moreover, we demonstrated that EscP interacts with the gatekeeper SepL and that their interaction is calcium dependent. On the basis of our results, we propose a molecular model to explain the second substrate switching event of the T3SS, which occurs in response to the formation of a continuous channel from the bacterial cytosol to that of the host cell upon host cell contact.

## RESULTS

### Calcium-sensing switch in EPEC.

It has previously been shown that the T3S of translocator and effector proteins by EPEC and other pathogens is altered by changes in the extracellular calcium concentration (32, 34, 41, 43, 44). It was suggested that by monitoring the calcium concentration next to the T3SS, the bacteria can distinguish between an assembling injectisome, which is exposed to a high extracellular calcium concentration, and a fully assembled T3SS, which is directly connected to the intracellular host cytosol, where calcium is very limited. Therefore, it was hypothesized that the T3SS possesses a mechanism to sense the reduction of calcium and to switch substrate secretion accordingly.

**FIG 1:**
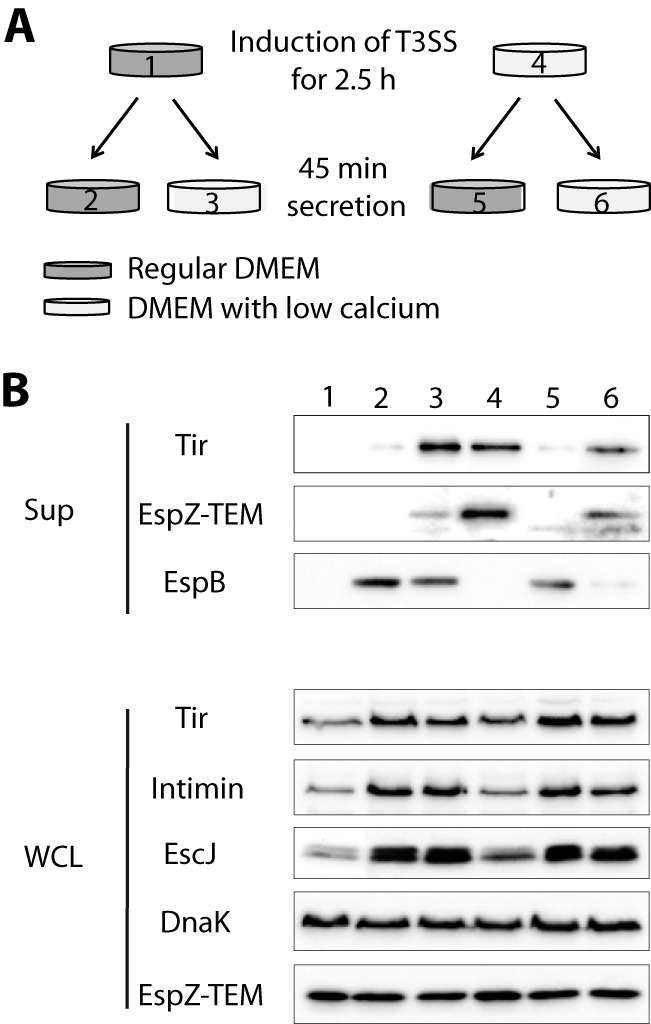
Extracellular calcium spike reversibly alters the secretion profile. A WT EPEC strain expressing the EspZ-TEM fusion protein was grown under T3S-inducing conditions for 2.5 h in regular (lane 1) or low-calcium (lane 4) DMEM. Protein expression (WCL) and secretion (Sup) of effectors and translocators, which were concentrated from supernatants of bacterial cultures, were detected with antibodies specific for EspB (translocator), Tir (effector), and EspZ-TEM (effector). After 2.5 h of induction, effector proteins were detected in the secretion fraction of bacteria grown in low-calcium DMEM (lane 4) but not in regular DMEM (lane 1). The translocator EspB was not found in the secreted fraction after 2.5 h, regardless of the growth conditions. Switching WT EPEC from regular DMEM to low-calcium DMEM resulted in greater secretion of effectors and lower levels of translocators (lane 3) than in bacteria that were continuously grown in regular DMEM (lane 2). Exposure of the bacteria grown in low-calcium DMEM to a calcium spike for 45 min resulted in a dramatic reduction of effector secretion and elevation of translocator secretion (lane 5 versus lane 6). These results indicated that the switch between translocator and effector secretion is reversible and strongly influenced by the extracellular calcium concentration. The expression of T3SS components, however, was not affected by the extracellular calcium concentration, as was demonstrated in WCL (lanes 1 to 6). To demonstrate the equal loading of lysates, the WCL was blotted with an anti-DnaK antibody.

To determine whether the switching event is permanent or reversible, we grew a WT EPEC strain transformed with an EspZ-TEM expression vector grown under T3SS-inducing conditions containing either a high CaCl_2_ concentration (1.8 mM) or a low calcium level (120 nM, similar to the calcium concentration present in the cytosol of host cells) for 2.5 h. Bacteria were then washed and moved to Dulbecco’s modified Eagle’s medium (DMEM) with either a high (regular DMEM) or a low CaCl_2_ concentration for an additional 45 min (Fig. 1A).

Samples of the secreted fractions (Sup) and of whole-cell lysates (WCL) were subjected to SDS-PAGE and examined by Western blot analysis for translocator and effector protein secretion (Fig. 1B). Subsequent normalization according to the bacterial optical density at 600 nm (OD_600_) showed that bacterial growth was not significantly affected by calcium concentrations (data not shown). Our results indicated that while the expression levels of the T3S proteins were not affected by calcium concentrations, the amounts of translocator and effector proteins secreted into the supernatants responded to the changes in calcium concentrations. The Tir effector, which is the first effector secreted, and the EspZ effector that is secreted later in the process were observed in the supernatant after 2.5 h of growth in low-calcium DMEM (lane 4), but they were absent from the parallel supernatant samples obtained after growth in high-calcium DMEM (lane 1). The translocator, EspB, was not detected in the supernatant samples after 2.5 h of growth in the presence or the absence of calcium. These results are consistent with a previous report (41).

The WT EPEC strain initially grown in high-calcium DMEM was transferred to a high-calcium (lane 2) or low-calcium (lane 3) medium. The sample transferred to the low calcium concentration showed increased secretion of effectors and reduced secretion of the translocator EspB. These results confirmed that a drop in the calcium concentration results in a shift from translocator secretion to the secretion of effectors. The experiment was then performed in the reverse order; i.e., the WT EPEC strain was grown first in low-calcium DMEM and then transferred to either a high (lane 5) or a low (lane 6) calcium concentration. Analyses of the supernatants of the sample exposed to the increased calcium concentration (lane 5) showed lower secretion of effectors and elevated translocator levels than those of the sample in which the calcium concentration was constant (lane 6). Overall, our results demonstrate that the translocator/effector switch is reversible and that switching occurs rapidly in response to changes in the calcium concentration.

### Identifying the proteins involved in calcium sensing.

The ability of EPEC to switch its secretion of substrates from translocators to effectors upon a drop in the calcium concentration was previously suggested to involve two T3SS proteins, SepL and SepD. EPEC Δ*sepL* and Δ*sepD* mutant EPEC strains were found to be calcium blind, meaning that their T3S profiles, showing hypersecretion of effectors, are identical regardless of the extracellular calcium concentration in the growth medium (34). However, it was suggested that an additional component of the T3SS is likely involved, since calcium concentrations do not affect SepD-SepL interaction (34). Accordingly, we focused on the involvement of the EscP protein in the calcium-sensing mechanism based on the altered translocator/effector secretion profile observed for the Δ*escP* (previously called orf16) mutant strain of *Citrobacter rodentium*, a murine pathogen that has a conserved T3SS in common with human EPEC and enterohemorrhagic *E. coli* (EHEC) (40).

**FIG 2:**
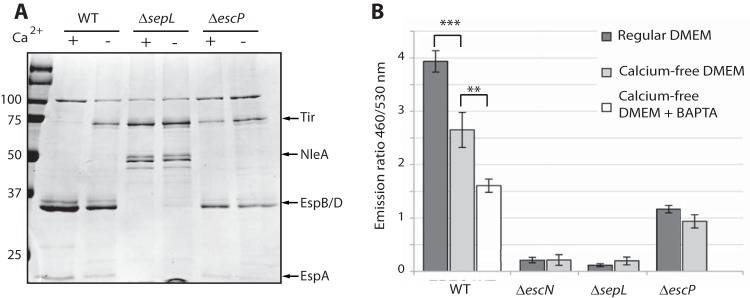
Calcium affects the secretion profile and infection rates of EPEC strains. (A) Protein secretion profiles of WT, *ΔsepL*, and *ΔescP* mutant EPEC strains grown in regular and calcium-free DMEM. The secreted proteins were concentrated from supernatants of bacterial cultures and analyzed by Coomassie staining after SDS-PAGE. Both mutant strains showed no sensitivity to the calcium concentration in the growth medium. The effectors (Tir, NleA) and translocators (EspB, EspD, EspA) are indicated by arrows. The values to the left are molecular sizes in kilodaltons. (B) Translocation assay for WT and *ΔescN* (a T3SS ATPase mutant), *ΔsepL*, and *ΔescP* mutant EPEC bacterial strains carrying the EspZ–TEM-1 fusion construct. The strains were grown in regular or calcium-free DMEM. HeLa cells were infected for 1.5 h and loaded with the β-lactamase substrate CCF2/AM, and fluorescence was measured with a microplate reader. Fluorescence quantification data, representing EspZ-Bla translocation, are presented as the ratio of the fluorescence emission of TEM β-lactamase-cleaved CCF2 (460 nm) to that of the original substrate (530 nm). Statistical significance was determined by Student’s *t* test (**, *P* < 0.001; ***, *P* < 0.0005).

To investigate the role of EscP in the regulation of translocator and effector secretion and its sensitivity to calcium concentrations, we generated a Δ*escP* null EPEC mutant and examined its T3SS activity. Similar to the results obtained by Monjarás Feria et al., the Δ*escP* mutant EPEC strain grown in regular DMEM (1.8 mM CaCl_2_) showed lower translocator and higher effector secretion levels than WT EPEC (Fig. 2A) (42). Interestingly, the secretion profile of the *escP* null mutant in regular DMEM with 1.8 mM CaCl_2_ was strikingly similar to that of WT EPEC grown in calcium-free DMEM. Moreover, the *ΔescP* mutant strain was found to be calcium blind (Fig. 2A). In agreement with previously reported results, the *ΔsepL* mutant strain exhibited hypersecretion of effectors but no secretion of translocators (Fig. 2A) (34). To correlate the T3SS activity of the mutant strains with their ability to translocate effectors into host cells, we performed the translocation assay developed by Charpentier et al. (45). In brief, HeLa cells were infected for 90 min with EPEC strains carrying an EspZ–TEM-1 fusion construct that were grown in either DMEM or calcium-free DMEM. At the end of the incubation, cells were washed and stained with CCF2/AM. Uninfected HeLa cells appeared green after incubation with CCF2/AM, indicating the absence of β-lactamase activity, while WT EPEC-infected HeLa cells appeared blue because of CCF2 cleavage by the EspZ-TEM-1 chimeric protein translocated into the host cells. WT EPEC grown in a calcium-free medium exhibited a lower effector translocation level than the same strain grown in regular DMEM (Fig. 2B). The addition of the calcium chelator 1,2-bis(*o*-aminophenoxy)ethane-*N*,*N*,*N*′,*N*′-tetraacetic acid (BAPTA) to the infection medium to remove any excess calcium further reduced the translocation level. The SepL null mutant exhibited very low translocation ability, regardless of the calcium concentration in the extracellular medium, probably because of its inability to secrete translocators. The EscP null mutant showed reduced translocation ability that was not dependent on the calcium levels found in DMEM and was comparable to the translocation activity of the WT strain grown in the absence of calcium and in the presence of BAPTA. On the basis of these results, we conclude that EscP is involved in the EPEC T3SS calcium-sensing mechanism.

### EscP is primarily involved in the blocking of effector secretion.

**FIG 3:**
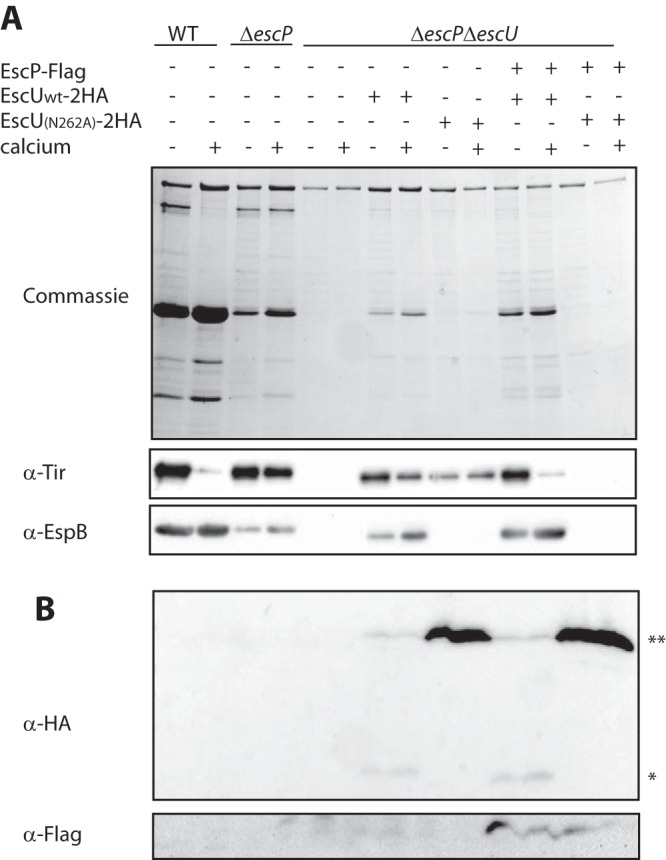
EscP is primarily involved in blocking of effector secretion. (A) Protein secretion profiles of WT and *ΔescP* and *ΔescP ΔescU* mutant EPEC strains transformed with a vector encoding either EscU_wt_ or EscU_N262A_ in the presence or absence of EscP-Flag and grown in regular or calcium-free DMEM. The secreted proteins were concentrated from supernatants of bacterial cultures and analyzed by Coomassie staining after SDS-PAGE or Western blot analysis (with anti-Tir or anti-EspB antibodies). (B) WCL were analyzed by Western blotting with antibodies specific to Flag and HA sequences. Full-length (**) and cleaved (*) forms of the EscU_wt_-2HA protein were detected, while only the full-length form of the EscU_N262A_-2HA noncleavable mutant protein was detected.

To distinguish between the effect of EscP on the secretion of translocators versus its effect on the secretion of effectors, we created a Δ*escP* Δ*escU* double deletion mutant and examined its secretion pattern when it was complemented with either WT EscU or a mutant form (N262A) that cannot switch from the early substrates to either the intermediate or the late substrate (20). As expected, the double deletion mutant had no T3SS activity but was complemented with the EscU_wt_ construct to a secretion pattern similar to that of the Δ*escP* mutant strain (Fig. 3A). Interestingly, transformation of the Δ*escP* Δ*escU* mutant EPEC strain with an expression vector containing the *escU*_N262A_ mutant gene produced elevated secretion of Tir (late substrates) but no secretion of translocators (intermediate substrates; EspB) (Fig. 3A). These results suggest that EscP is primarily involved in the blocking of effector secretion and has only a secondary effect on translocator secretion. Complementation of the double deletion strain with EscP-Flag (which functionally complements the Δ*escP* mutant; see Fig. S1 in the supplemental material) and with EscU_wt_ complemented its calcium sensitivity, but no T3S activity was observed when the same strain was complemented with EscP-Flag and EscU_N262A_, as previously reported for the Δ*escU* mutant strain transformed with the EscU_N262A_ vector (20).

10.1128/mBio.01733-16.1 Figure S1  Flag-tagged EscP can complement the Δ*escP* mutant. Shown are protein secretion profiles of WT and Δ*escN*, Δ*escP*, and Δ*escP* mutant EPEC strains complemented with pEscP-Flag strains grown under T3SS-inducing conditions. Secreted proteins were concentrated from supernatants of bacterial cultures and analyzed by SDS–12% PAGE and Coomassie blue staining. The locations of type III secreted proteins EspA, EspB, EspD, and Tir are indicated to the right of the gel. Also indicated is the location of EspC, which is not secreted via the T3SS. We observed that deletion of the *escP* gene results in the unregulated secretion of translocator and effector proteins, but transformation of the mutant with the pEscP-Flag vector fully restored T3SS secretion regulation. Similar results were obtained for an EscP protein labeled with a double HA tag (2HA; data not shown) and are consistent with previously reported data (42). Download Figure S1, EPS file, 2.2 MB. Copyright © 2017 Shaulov et al. 2017 Shaulov et al. This content is distributed under the terms of the Creative Commons Attribution 4.0 International license.

### EscP contains a secretion signal and localizes to the inner membrane (IM).

On the basis of its involvement in the control of T3SS needle length, EscP was previously suggested to function as a ruler protein in EPEC, where it is secreted at low levels by the T3SS. EscP secretion was reported to occur constitutively and independently of the T3SS (42). This is in marked contrast to the ruler proteins of other pathogens, such as *Salmonella*, *Yersinia*, and *Shigella* spp., which are secreted only in a T3SS-dependent manner (21, 46–48). To clarify whether and how EscP is secreted and to identify its secretion signal, we generated various fusions of the EscP protein (full length and the two N-terminal amino acid sequences comprising residues 1 to 20 and 1 to 50) with the reporter protein β-lactamase (TEM-1) and evaluated the extent of their secretion into the extracellular medium under T3SS-inducing conditions. The chimeric proteins were expressed in WT EPEC (normal secretion), the Δ*escN* mutant (no secretion), and the Δ*sepD* and Δ*sepL* mutants (hypersecretion of effectors). Because many T3SS proteins containing a secretion signal exhibit significantly higher secretion in the Δ*sepD* and Δ*sepL* mutant strains (37, 49), we used these strains to enable the detection of low levels of secreted substrates.

**FIG 4:**
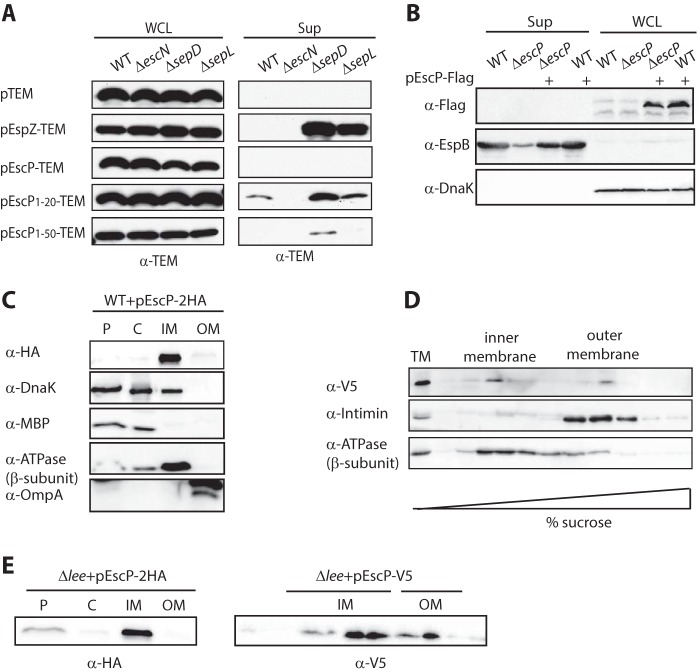
EscP has a secretion signal but is not secreted through the T3SS. (A) Western blotting against TEM of WCL and secreted fractions (Sup) of WT and Δ*escN*, Δ*sepD*, and Δ*sepL* mutant EPEC strains expressing TEM, EspZ-TEM, EscP-TEM, and two N-terminal regions of EscP (EscP_1–20_ and EscP_1–50_) fused to TEM protein grown in DMEM under T3S-inducing conditions. The N-terminal amino acid sequence of EscP containing residues 1 to 20 functions as a secretion signal, but it is concealed by the sequence immediately after it. (B) WT and Δ*escP* mutant EPEC strains were transformed with a plasmid carrying EscP fused to the Flag tag and grown under T3SS-inducing conditions. WCL and secreted (Sup) fractions were analyzed by Western blotting with antibodies specific to Flag and EspB. Samples were also probed with an anti-DnaK antibody to demonstrate equal loading of lysates and to exclude the possibility of cell lysis during the experiment. (C) A WT EPEC strain expressing EscP-2HA was grown under T3S-inducing conditions and fractionated into periplasmic (P), cytoplasmic (C), IM, and OM fractions. The samples were separated by SDS–16% PAGE and analyzed by Western blotting with anti-HA antibody. To confirm correct bacterial fractionation, the Western blots were probed with anti-DnaK (cytoplasmic marker), anti-MBP (periplasmic marker), anti-ATPase β subunit (IM marker), and anti-OmpA (OM marker) antibodies. (D) The total membrane (TM) fraction was isolated from the Δ*escP* mutant complemented with pEscP-V5, applied on the top of a sucrose density gradient, and subjected to ultracentrifugation at 210,000 × *g* for 14 h. Fractions were collected and concentrated, and aliquots were separated by SDS–16% PAGE, followed by Western blot analysis with anti-V5 antibody. As controls for IM and OM proteins, we used anti-ATPase β subunit and anti-intimin antibodies, respectively. (E) A Δ*lee* mutant EPEC strain expressing EscP-2HA was grown and fractionated as described for panel C (left), and the Δ*lee* mutant strain expressing EscP-V5 was fractionated as described for panel D (right).

All of the chimeric proteins exhibited similar expression levels in WCL (Fig. 4A). Active secretion, however, was detected only in the positive control, i.e., the EspZ effector fused to TEM-1, and in the chimeric protein containing the initial 20-amino-acid N-terminal sequence of EscP fused to TEM-1. No active secretion of full-length EscP fused to TEM-1 was detected, even when the chimeric proteins were expressed in the *ΔsepD* or *ΔsepL* mutants. On the basis of these results, we hypothesize that EscP has a secretion signal localized in its N-terminal region and an additional inhibitory sequence located between the amino acids at positions 20 to 50, which prevents active secretion through the T3SS (Fig. 4A). To determine whether the observed inconsistency in the ability of T3SS to secrete EscP is due to fusion of the latter to the relatively large reporter protein TEM-1, we examined the secretion of EscP tagged with the small Flag tag, which was shown to complement the Δ*escP* mutant (see Fig. S1 in the supplemental material). Western blot analysis with the anti-Flag antibody detected the EscP protein in the WCL but not in the secreted fraction of either the WT or the *ΔescP* mutant EPEC strain (Fig. 4B). These results suggest that although EscP contains a secretion signal, it is not secreted through the T3SS, and thus, its function occurs inside the bacteria.

A previous study found that YscP, a homolog of EscP in *Yersinia enterocolitica*, is located at the cell surface and is released when calcium ions are chelated (50). To determine whether EscP changes its localization in response to the calcium concentration, we examined whether EscP is secreted at low extracellular calcium concentrations. Tests of EscP secretion both in regular DMEM and in calcium-free DMEM when expressed in WT and Δ*escP* mutant EPEC strains did not detect EscP secretion under either condition (see Fig. S2 in the supplemental material). We therefore conclude that although both YscP and EscP are calcium sensitive, calcium ions affect the two proteins differently.

10.1128/mBio.01733-16.2 Figure S2  EscP is not secreted into calcium-free medium. WT and Δ*escN* and Δ*escP* mutant EPEC strains expressing EscP-Flag were grown under T3SS-inducing conditions in either regular or calcium-free DMEM. WCL and secreted proteins were separated by SDS–14% PAGE and analyzed by Western blotting with an antibody against Flag and antibodies specific to T3SS components. WCL was probed with an anti-DnaK antibody to demonstrate equal loading of lysates. Download Figure S2, EPS file, 2.9 MB. Copyright © 2017 Shaulov et al. 2017 Shaulov et al. This content is distributed under the terms of the Creative Commons Attribution 4.0 International license.

To determine whether the EscP protein is cytosolic, as predicted by its sequence, or membrane associated, we examined its subcellular localization in WT EPEC by using EscP labeled with a double hemagglutinin (HA) tag at its C terminus. Bacteria were grown under T3SS-inducing conditions, and whole-cell extract was fractionated into cytoplasmic, periplasmic, and IM and outer membrane (OM) fractions. Western blot analysis with an anti-HA antibody revealed that EscP-2HA was localized mainly to the IM (Fig. 4C). To confirm that EscP localization to the IM fraction was not a result of protein overexpression and aggregation, we subjected the membrane fraction of the EPEC strain encoding EscP in a low-copy-number expression plasmid to a sucrose density gradient assay (30 to 55% sucrose). Intimin and the β subunit of ATPase were used as markers for the OM and IM fractions, respectively. Similar to the β subunit of ATPase, EscP-V5 was found to localize mainly to the IM fractions (Fig. 4D), further supporting the IM localization of EscP despite predictions that it is a soluble protein. To determine whether EscP localization is T3SS dependent, we introduced the EscP-2HA or EscP-V5 plasmids into the Δ*lee* mutant EPEC strain, which lacks the entire pathogenicity island and any T3SS structural components. We found that EscP was still localized mostly to the IM, suggesting that its localization is T3SS independent (Fig. 4E). The IM localization of EscP was examined and found to be insensitive to the calcium concentration (data not shown).

### EscP interacts with SepL but not with SepD, and the binding of EscP and that of SepD to SepL are mutually exclusive.

**FIG 5:**
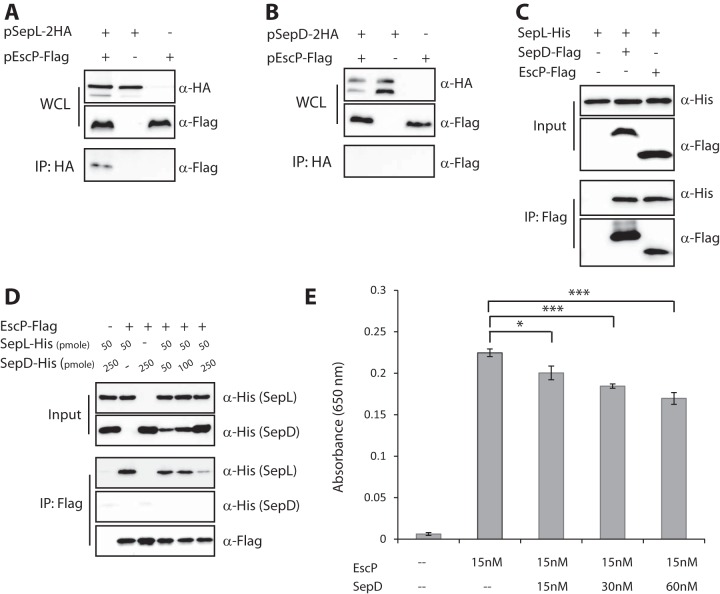
EscP interacts with SepL but not with SepD. A WT EPEC strain was transformed with pSepL-2HA (A) or pSepD-2HA (B) with or without pEscP-Flag. The bacteria were grown under T3S-inducing conditions. Whole-cell extracts were immunoprecipitated (IP) with an antibody against HA. WCL and eluted samples were analyzed by Western blot assay probing for EscP (with anti-Flag antibody) and SepL or SepD (with anti-HA antibody). EscP coprecipitated with SepL but not with SepD. (C) WCL of *E. coli* BL21(λDE3) transformed with SepD-Flag or EscP-Flag were incubated with protein G slurry beads bound to the anti-Flag antibody. The beads were then washed and exposed to WCL of *E. coli* BL21 lysate expressing SepL-His. WCL and eluted samples were analyzed by Western blotting with anti-Flag and anti-His antibodies. The left lane, which lacks a Flag-tagged protein, served as a negative control. (D) WCL of *E. coli* BL21(λDE3) expressing the EscP-Flag protein were incubated with anti-Flag antibody and protein G slurry beads, and the beads were washed and incubated with purified SepL-His (50 pmol) and SepD-His at different concentrations (as shown). No Flag antibody was added to the reaction mixture in the left lane. (E) ELISA-based analysis of the interaction between the purified SepL and EscP proteins in the absence or presence of increasing amounts of SepD protein (15, 30, and 60 nM). Histograms of the mean absorbance at 650 nm with standard deviations are plotted. Statistical significance was determined by Student’s *t* test (*, *P* < 0.005; ***, *P* < 0.0005).

To determine the role of EscP in the calcium-sensing mechanism, we examined whether EscP can interact with either substrate hierarchy switch protein SepL or SepD. Although these two proteins were shown to interact with each other (34, 38, 40), their ability to bind EscP has not been reported. To study protein interactions, HA-tagged SepL or SepD was transformed into WT EPEC alone or in the presence of EscP-Flag. Immunoprecipitation with an anti-HA antibody showed that EscP coeluted with SepL-2HA (Fig. 5A) but not with SepD-2HA (Fig. 5B). Strains carrying one of the proteins were used as negative controls to examine nonspecific interactions. Purified SepL-His protein pulldown experiments demonstrated that it interacts with EscP-Flag in EPEC lysates (data not shown), further supporting our initial results regarding the SepL-EscP interaction. Overall, on the basis of our experiments, we conclude that SepL, but not SepD, is a binding partner of EscP.

Next, we examined whether the interaction between SepL and EscP is direct or mediated by an additional T3SS component. To that end, we expressed the SepL, SepD, and EscP proteins in the *E. coli* BL21 strain, which does not contain a T3SS, and performed immunoprecipitation with an anti-Flag antibody. The experimental setup entailed coating the beads with either EscP-Flag or SepD-Flag and then incubating them with bacterial lysates expressing SepL-His. Using this approach, we observed interactions between SepL and EscP and confirmed the previously reported interactions between SepL and SepD (Fig. 5C). On the basis of these results, we conclude that the binding of EscP to SepL does not require additional T3SS components.

We then used a co-IP assay to investigate the ability of the three proteins to form a protein complex. Bacterial lysates containing EscP-Flag were subjected to immunoprecipitation with anti-Flag antibody, and the beads were washed and then incubated with either 50 pmol of purified SepL-His (lane 2), 250 pmol of purified SepD-His (lane 3), or combinations of 50 pmol SepL-His in the presence of increasing amounts of SepD-His (50, 100, and 250 pmol). A negative control, which did not contain the anti-Flag antibody, confirmed that neither SepL-His nor SepD-His can bind nonspecifically to the beads (Fig. 5D, lane 1). As expected, SepL-His, but not SepD-His, coeluted with EscP-Flag (Fig. 5D, lanes 2 and 3). Interestingly, the addition of increasing amounts of purified SepD (50, 100 and 250 pmol) to the reaction mixtures containing 50 pmol of purified SepL and EscP interfered with the ability of EscP to coelute with SepL-His (Fig. 5D, lanes 4 to 6), suggesting that SepD competes with EscP for binding to SepL. Moreover, these results suggest that SepL can form two protein complexes: SepL/SepD and SepL/EscP. An enzyme-linked immunosorbent assay (ELISA)-based analysis with purified proteins confirmed that SepL and EscP interact directly and that SepD competes with EscP in interactions with SepL (Fig. 5E). Because of the high levels of SepD and SepL protein expression, compared to that of EscP (data not shown), in WT EPEC, we propose that during T3S both the SepL-SepD and SepL-EscP complexes are present (see Fig. 7B).

### Interaction between EscP and SepL is calcium dependent.

**FIG 6:**
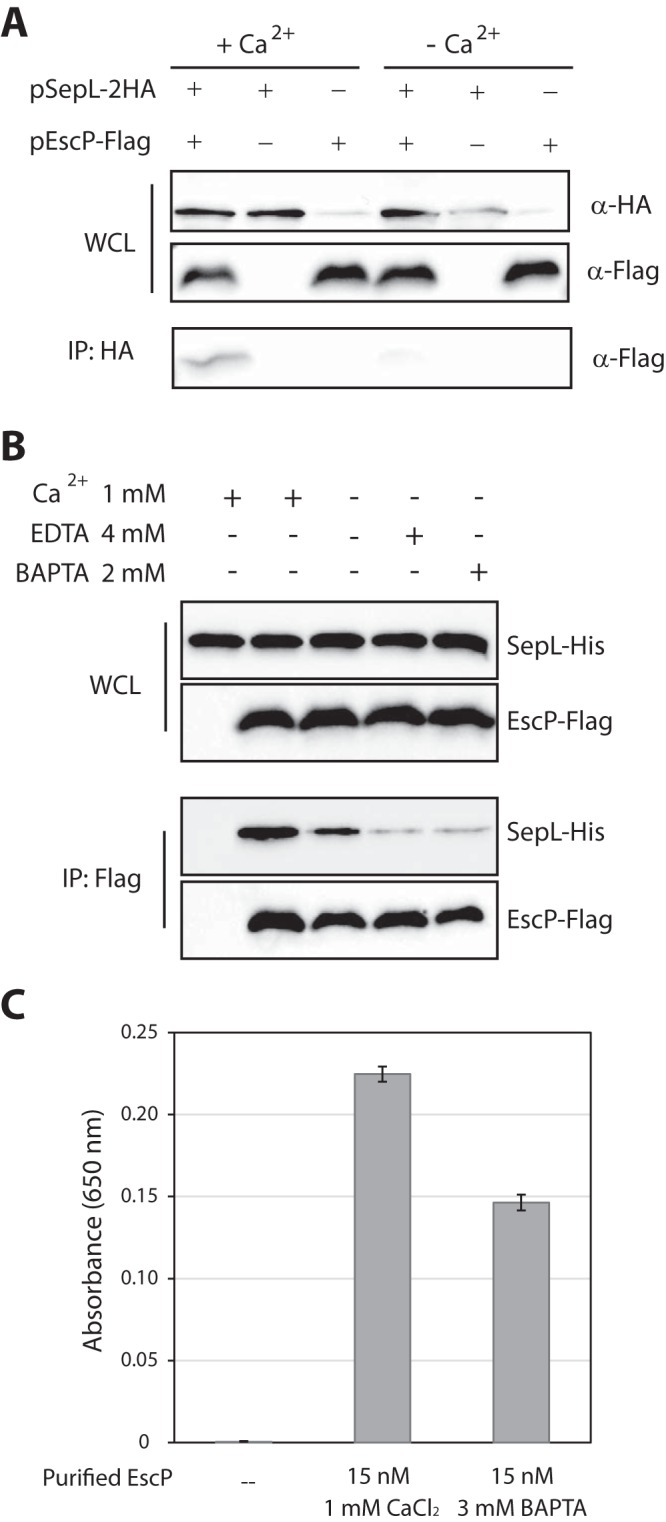
EscP interacts with SepL in a calcium-dependent manner. EscP-SepL *in vivo* (A) and *in vitro* (B) interactions were performed as described for Fig. 5A and C, respectively. To determine the involvement of calcium in stabilization of the interactions, the lysis and wash buffers used for the experiments were either calcium free (−Ca^2+^) or contained 2 mM CaCl_2_ (+Ca^2+^). In the *in vitro* experiments (B), two samples each contained a chelator agent, BAPTA or EDTA, as indicated. (C) ELISA-based analysis of the interaction between purified SepL and EscP in the presence of calcium or a calcium chelator (BAPTA). Histograms show the mean absorbance at 650 nm with standard deviations.

To determine the effect of calcium on EscP-SepL interaction, we performed coimmunoprecipitation (co-IP) experiments with an anti-HA antibody. WT EPEC transformed with EscP-Flag, SepL-2HA, or both was grown under T3SS-inducing conditions, collected, washed, and resuspended in a buffer with or without 2 mM CaCl_2_. Reduced levels of coeluted EscP-Flag were obtained with bacterial lysates that contained no calcium (Fig. 6A), indicating that the EscP-SepL interaction is calcium dependent. Moreover, lower levels of EscP-Flag coeluted after addition of the specific calcium chelator BAPTA to the resuspension buffer (see Fig. S3A in the supplemental material).

10.1128/mBio.01733-16.3 Figure S3  EscP interacts with SepL in a calcium-dependent manner. (A) To examine whether the EscP-SepL interaction is calcium dependent *in vivo*, WT EPEC transformed with a plasmid encoding SepL-2HA, EscP-Flag, or both was subjected to a co-IP experiment with an anti-HA antibody. The lysis buffer was supplemented with 2 mM CaCl_2_ or BAPTA. WCL and eluted samples were subjected to SDS–16% PAGE and probed with an antibody to the Flag or HA tag. (B) To reveal whether the SepL-EscP interaction is calcium sensitive *in vitro*, *E. coli* BL21 coexpressing EscP-Flag and SepL-His or EscP-Flag and Sumo-His was subjected to co-IP experiments with an anti-Flag antibody. The lysis buffer was supplemented with 2 mM CaCl_2_, 4 mM EDTA, or 2 mM BAPTA. Eluted samples were subjected to SDS–16% PAGE and probed with an anti-His or anti-Flag antibody. Download Figure S3, EPS file, 2.8 MB. Copyright © 2017 Shaulov et al. 2017 Shaulov et al. This content is distributed under the terms of the Creative Commons Attribution 4.0 International license.

Next, we examined the calcium sensitivity of the EscP-SepL interaction to determine whether it is conserved *in vitro* or requires additional T3SS components. Co-IP experiments were done with cell lysates of BL21 strain expressing EscP-Flag and SepL-His. Samples that contained EDTA or BAPTA showed significantly lower levels than the calcium-containing sample (Fig. 6B), confirming that the calcium dependence of the EscP-SepL interaction is not dependent on any additional T3SS components. We further validated these results by ELISA (Fig. 6C) and a pulldown experiment (see Fig. S3B in the supplemental material). In addition, we performed several calcium binding experiments to elucidate whether the EscP protein binds calcium, but those results were inconclusive (data not shown). Finally, in line with a previous report, we found that calcium does not affect the interaction between SepL and SepD under conditions similar to those used to examine the SepL-EscP interaction (see Fig. S4 in the supplemental material) (34).

10.1128/mBio.01733-16.4 Figure S4  SepL-SepD interaction is not calcium sensitive. A WT EPEC strain was transformed with a plasmid encoding SepL-2HA and EscP-Flag (A), pSepL-2HA and SepD-Flag (B), or pSepL-2HA and SepD-V5 (C). Panel A was used as a positive control, while panel C was used to exclude the possibility that the tag attached to the protein affects calcium sensitivity. Co-IP experiments were performed with an anti-HA antibody. The lysis buffer was supplemented with 2 mM CaCl_2_ or BAPTA. Lane 1 in every panel lacked the anti-HA antibody and therefore served as a negative control. Eluted samples were subjected to SDS–16% PAGE and probed with an anti-Flag or anti-V_5_ antibody to detect the coprecipitation of EscP or SepD. Download Figure S4, EPS file, 2.8 MB. Copyright © 2017 Shaulov et al. 2017 Shaulov et al. This content is distributed under the terms of the Creative Commons Attribution 4.0 International license.

## DISCUSSION

Calcium was previously suggested to be involved in the regulation of type III substrate secretion, primarily in the switch from intermediate to late substrates (32, 34). However, it was not clear whether calcium is the actual physiological signal sensed by the bacteria, as no direct link between T3SS proteins and calcium was found. In this study, we identified a T3SS protein complex that is sensitive to calcium concentrations and that dissociates upon chelation of calcium. We propose that this complex is involved in blocking of the secretion of effectors until the assembly of the T3SS is complete and the calcium concentration at the base of the T3SS drops.

The protein secretion profiles of many Gram-negative pathogens, such as EHEC, EPEC, *Vibrio* and *Yersinia* spp., were shown to change in response to extracellular calcium concentrations (28, 34, 51). Mutant strains, which were found to be calcium blind *in vitro*, were attenuated *in vivo*, providing a strong indication that calcium sensing is an essential virulence determinant (40). In this study, we found an additional calcium-blind mutant EPEC strain, Δ*escP*, whose T3S profile is similar regardless of the extracellular calcium concentration. In contrast to the two previously known calcium-blind Δ*sepL* and Δ*sepD* mutant EPEC strains, the Δ*escP* mutant strain demonstrated a T3S profile identical to that of the WT EPEC strain grown in calcium-free medium (Fig. 2), suggesting that EscP is the main protein involved in the mechanism of substrate switching in response to calcium.

**FIG 7:**
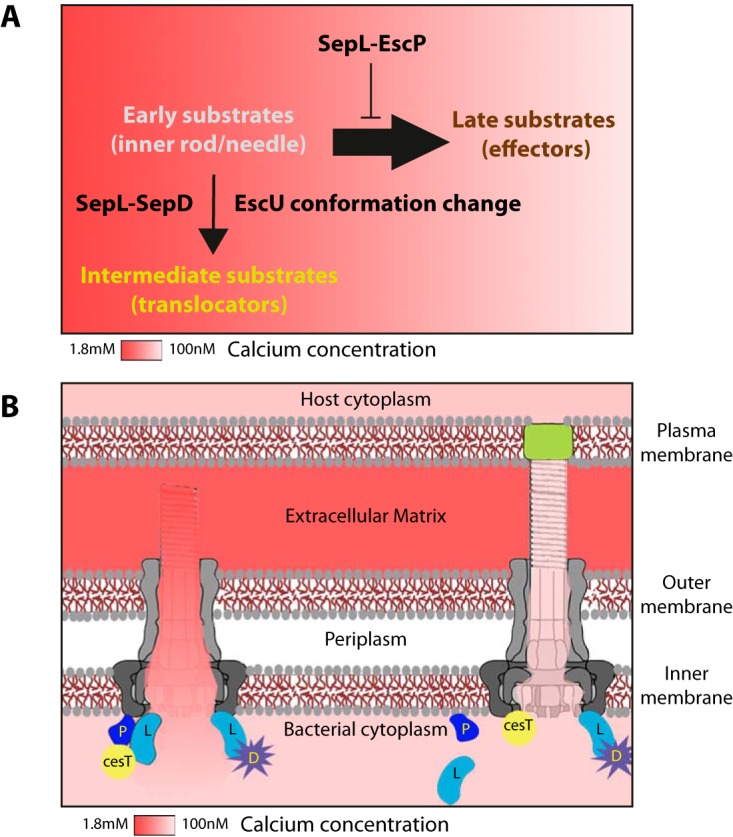
Schematic representation of the SepL-EscP calcium-sensing mechanism. (A) The substrate secretion hierarchy of early, intermediate, and late substrates is not sequential. Rather, the process proceeds directly from early to late. The transient divergence to secrete intermediate substrates happens when actively promoted by SepL-SepD complex formation and after EscU undergoes a conformational change due to an autocleavage event. Effector secretion is delayed until the SepL-EscP complex dissociates (upon a drop in the calcium concentration). (B) Intermediate substrates are secreted while the T3SS filament is being assembled and extracellular calcium can leak into the bacteria through the incomplete channel. This increases the local calcium concentrations at the base of the T3SS, which stabilizes the interaction between SepL (light blue, L) and EscP (dark blue, P). Binding of the complex to CesT (yellow) inhibits effector secretion. Upon formation of the pore complex (light green) within the host membrane, the calcium concentration in the hollow conduit and at the base of the T3SS drops, thereby inducing SepL-EscP complex dissociation and initiation of effector secretion. The SepL-SepD complex is not affected by the calcium concentration.

The secretion profile of the Δ*escP* mutant EPEC strain showed that secretion of the inner rod protein (early substrate) (42) and of the effector proteins (late substrates) was elevated, while that of translocators (intermediate substrates) was reduced (Fig. 2A and 3A). These results indicate that the T3S process is most likely not a linear, stepwise process, i.e., one that proceeds sequentially from early to intermediate and then to late substrates. Instead, it switches directly from early to late substrates and secretes intermediate substrates only when promoted by the SepD-SepL complex and after the conformational change in EscU that occurs because of an autocleavage event (Fig. 7A). Moreover, this secretion of the intermediate substrates (translocators; EspA, EspB, and EspD in EPEC) requires both the inhibition of late substrate secretion and the promotion of translocator secretion (Fig. 7A). In light of the T3S profiles of the Δ*escP*, Δ*sepL*, Δ*sepD*, and Δ*escP* Δ*escU* mutant strains, we suggest that translocator secretion requires the coordinated activity of EscP, SepL, SepD, and EscU autocleavage. This is based on our observations that translocator secretion is largely reduced in the absence of EscP and completely eliminated in the absence of the SepL and SepD proteins and in the presence of the EscU_N263a_ mutant form. The SepL-SepD complex is essential for the secretion of translocators, while the SepL-EscP complex has a regulatory effect that prevents premature effector secretion and therefore has an indirect effect on the secretion of translocators. The secretion of Tir, observed for the Δ*escP* Δ*escU* EPEC strain transformed with an expression vector encoding the *escU*_N262A_ mutant gene (Fig. 3) further supports our suggestion that the EscP-SepL protein complex plays a direct role in inhibiting the premature secretion of effectors and that it is not directly involved in translocator secretion.

In this study, we found that although the SepL, SepD, and EscP proteins are involved in promotion of the secretion of translocators, two different complexes are formed: SepL-SepD (34) and SepL-EscP (Fig. 5). The SepL-SepD complex is essential for the secretion of translocators, and therefore, deletion of either the *sepL* or the *sepD* gene results in the complete elimination of translocator secretion. The SepL-EscP complex, on the other hand, inhibits premature effector secretion before the translocon complex is fully assembled. Our results are in agreement with those of a previous study in which it was observed that YopN, the *Yersinia* homolog of SepL, participates in two different complexes, one that promotes and another that blocks secretion (50).

Our finding that SepL-EscP interaction is calcium sensitive establishes a link between an environmental signal in the form of calcium and the regulation of secretion substrate switching. Although calcium was previously considered to be a strong candidate for a signal molecule that indicates the formation of a hollow conduit connecting the bacteria to the host cell cytoplasm, its exact role in host sensing and T3SS substrate regulation has never been reported. The secretion profiles of the EPEC WT strain initially grown in a high calcium concentration and then transferred to growth medium with a low calcium concentration together with the profiles of the reverse experiment demonstrated that the response to calcium alteration is rapid and reversible, suggesting that calcium regulation happens at the protein level (Fig. 1).

On the basis of our results, we propose the following model as a substrate-switching mechanism. During T3SS filament assembly, extracellular calcium can leak into bacteria through the incomplete channel and increase the local calcium concentration at the base of the T3SS. This stabilizes the SepL-EscP complex, thereby inhibiting effector secretion. Upon formation of the pore complex within the host membrane, the calcium concentration in the hollow conduit and at the base of the T3SS drops. The reduction in the calcium concentration induces SepL-EscP complex dissociation and the initiation of effector secretion. On the basis of previous reports that EscP and SepL can bind Tir and CesT, the chaperone of Tir, we hypothesize that CesT/Tir binds the SepL-EscP complex (42, 52). While bound to that complex, CesT/Tir is denied access to the secretion platform, which delays the secretion of Tir. Once the SepL-EscP complex dissociates, CesT-Tir can dock at the secretion platform and initiate effector secretion (Fig. 7B).

According to this model, the substrate switching mechanism does not require that EscP be actively secreted through the T3SS, as was suggested for the ruler protein model (10, 12, 53). In our study, no full-length EscP protein secretion was detected, a finding that was supported by a previous study that analyzed the EPEC secretome by stable isotope labeling with amino acids in cell culture and did not find the EscP protein in the EPEC secretome of either WT EPEC or of the Δ*sepD* mutant (49). We speculate that the role of the type III secretion signal carried by EscP at its N terminus (Fig. 4) is to target EscP to the T3SS apparatus, where EscP can exert its regulatory function. An examination of EscP secretion under low-calcium conditions, which were found to induce YscP secretion (48), concluded that full-length EscP is not secreted when cells are grown under calcium-free conditions (see Fig. S2 in the supplemental material). Although an earlier study by Monjarás Feria and colleagues reported EscP protein secretion (42), in that study, the researchers concluded that EscP secretion is constitutive and not T3SS dependent, suggesting that it is not part of the secretion regulation mechanism and that it may be an artifact of unique growth conditions.

In summary, the results of this study suggest that EscP is involved in the calcium-sensing mechanism that allows the T3SS of EPEC to switch substrate secretion from translocators to effector proteins upon the completion of T3SS translocon assembly and formation of the direct connection between the cytoplasm of the bacteria and the host cell. Because of the low level of conservation among the members of the YscP/InvJ/Spa32/FliK family of proteins, in terms of both protein sequence and characteristics (length, ability to be secreted, isoelectric point, etc.), further research is needed to determine whether the suggested switching mechanism and its link to calcium are mediated by all ruler proteins or only EscP.

## MATERIALS AND METHODS

### Bacterial strains and deletion mutants.

WT EPEC O127:H6 strain E2348/69 (streptomycin resistant) was used to assess the activity of the T3SS, *E. coli* strain DH10B was used for plasmid handling, and *E. coli* strain BL21(λDE3) was used for protein expression and purification. Bacteria were grown in Luria-Bertani (LB) broth supplemented with the appropriate antibiotics at 37°C with shaking or as static cultures, depending on the experimental requirements. Antibiotics were used at the following concentrations: streptomycin, 50 μg/ml; ampicillin, 100 μg/ml; carbenicillin, 100 μg/ml; kanamycin, 50 μg/ml; chloramphenicol, 34 μg/ml; tetracycline, 12.5 µg/ml.

### Construction of *escP*, *escP escU*, and whole *lee* null mutants.

Nonpolar deletion mutants of Sm^r^ EPEC strain E2348/69 were generated by the *sacB*-based allelic exchange method (54). Briefly, two PCR fragments were generated with the corresponding primer pairs (Table 1), cloned into pCR2.1-TOPO (Invitrogen), verified by DNA sequencing, and then subcloned as a KpnI/NheI fragment and a NheI/SacI fragment into a KpnI/SacI-digested suicide vector, pRE112 (55). The resulting plasmid, which contained the flanking regions of *escP* with about 70% of *escP* deleted, was transformed into *E. coli* SM10λ*pir* and then introduced into EPEC by conjugation. After sucrose selection, EPEC colonies that were resistant to sucrose and susceptible to chloramphenicol were screened for the deletion of *escP* by PCR. The Δ*escP* Δ*escU* double deletion mutant strain was created by a similar methodology by using an SM10λ*pir* strain containing a pRE112 vector designed for deletion of the *escU* gene (20) and introducing it into a Δ*escP* mutant EPEC strain. A whole *lee* deletion mutant was created by a similar methodology but with different PCR primer sets to delete the entire LEE pathogenicity island (Table 1).

**TABLE 1:** Sequences of the primers used in this study

Construct and primer	Primer sequence (restriction site)^a^
*escP* deletion mutant	
ESCP-01F	GCGGTACCGGCATAGGCAGACCAATGGAG (KpnI)
ESCP-01R	CCGCTAGCCAATCTCTTCCATAAAGAAGC (NheI)
ESCP-02F	GCGCTAGCAAGTTAAAAGAAATCTTTCCTG (NheI)
ESCP-02R	GCGAGCTCCGGAATTCACTAACTTGATGG (SacI)
*lee* deletion mutant	
LEE-01F	CGGCTAGCAAAAACCTGAATAACCCC (NheI)
LEE-01R	CGGAGCTCGATAGGTGGTATAGAATGCAGCAGCAAATG (SacI)
LEE-02F	GCGGTACCTGCCGTTCCTTAGTTCTGCACTTCTTG (KpnI)
LEE-02R	GCGCTAGCCATTTCCCGGCGTTACCATACG (NheI)
EscP-flag in pACYC184	
EscP-Flag-F	CCCGGATCCCTGCAACATGTTAATAAATC (BamHI)
EscP-Flag-R	CCCGTCGACTTATTTGTCATCGTCATCCTTGTAGTCTCCTCCATTTTCATATTCAATTGTGAAC (SalI)
EscP-V5 in pACYC184	
V5_Gib_F	GGTAAGCCTATCCCTAACCCTC
pACYC_Gib_R	TTTTAAATTTTATTCATCCTGGTGGTTG
EscP_V5_F	AGGATGAATAAAATTTAAAATGACTAGAGTTTCTCTAAAAAG
EscP_V5_R	GAGGGTTAGGGATAGGCTTACCTCCTCCATTTTCATATTCAATTG
EscP-2HA in pTOPO	
EscP-2HA-F	CCCGAGCTCCTGCAACATGTTAATAAATC (SacI)
EscP-2HA-R	CCTCGAGATTTTCATATTCAATTGTGAACTC (XhoI)
EscP-flag in pET21	
F_EscP_Flag_pet21	CCCATATGACTAGAGTTTCTC (NdeI)
R1_EscP_Flag	TCATCCTTGTAGTCTCCTCCATTTTCATATTCAA
R2_EscP_Flag	CCAAGCTTTATTTGTCATCGTCATCCTTGTAGTC (HindIII)
pGST-EscP-Flag in pGEX6	
F_EscP_Flag	CCGGATCCATGACTAGAGTTTCTC (BamHI)
R_GST_EscP_Flag	CCCTCGAGTTATTTGTCATCG (XhoI)
pSepL-His in pET28a	
F_sepL_His_pet28	CCCCATGGCTAATGGTATTG (NcoI)
R_sepL_His_pet28	CCCTCGAGCATAACATCCTCCTTATAAT (XhoI)
pSepD-His in pET21a	
F_SepD_pet21	CCCCATATGAACAATAATAATGGCATAGCAA (NdeI)
R_SepD_pet21	CCCTCGAGCATAACATCCTCCTTATAAT (XhoI)
pEscP-TEM	
EscP-TEMF	GCATATGACTAGAGTTTCTCTAAAAAG (NdeI)
EscP-TEMR	CGAATTCTCATTTTCATATTCAATTGTGAAC (*Eco*RI)
pEscP_1-20_-TEM EscP_1–20_-TEMR	CGAATTCTCATCTGATGGCCGAAAAGAAAC (*Eco*RI)
pEscP_1-50_-TEM EscP_1–50_-TEMR	CGAATTCTCAGAAGCCATTCGTATGAACAAC (*Eco*RI)
pSepD-V5 in pACYC184	
F_SepD_V5	CCGTCGACATGAACAATAATAATGGCATAGCAAAGAATG (SalI)
R1_SepD_V5	GGAGAGGGTTAGGGATAGGCTTACCCACAATTCGTCCTATA
R2_SepD_V5	CCGGATCCTTACGTAGAATCGAGACCGAGGAGAGGGTTAGGG (BamHI)

^a^ Restriction sites are underlined.

### Construction of plasmids expressing EscP, SepL, and SepD.

The *escP* gene was amplified from EPEC genomic DNA with primer pair EscP-F/EscP-R (Table 1), which fused a Flag sequence to the coding region. The PCR product was digested with BamHI/SalI and ligated into a BamHI/SalI-digested pACYC184 vector. The resulting plasmid expressed EscP fused to a Flag tag. EscP fused to the V5 tag was constructed by the Gibson assembly method (56). In brief, *escP* was amplified with primers EscP_V5_F/EscP_V5_R and the pACYC184 vector, which contained a V5 tag, was amplified with the V5_Gib_F/pACYC_Gib_R primer pair (Table 1). The PCR products were subjected to digestion with DpnI, purified, and assembled by the Gibson assembly method. The *escP* gene was also amplified with primer pair EscP-2HA-F/EscP-2HA-R (Table 1), cloned into pCR2.1-TOPO (Invitrogen), and then subcloned as a SacI/XhoI fragment into SacI/XhoI-digested pTOPO-2HA (40). The resulting construct, pEscP-2HA, expressed a fusion protein of EscP with a double HA tag at its C terminus.

Plasmid pCX341, a derivative of pCX340 with its origin of replication replaced with that of pBR322, was used to generate N-terminal translational fusions to the mature form of TEM-1 β-lactamase (45, 57). The coding regions of full-length EscP (without the stop codon) and two N-terminal amino acid sequences derived from EscP comprising residues 1 to 20 and 1 to 50 were amplified by PCR and cloned in front of *blaM* in pCX341 by using restriction enzymes NdeI and EcoRI. The resulting plasmids, pEscP-TEM, pEscP_1-20_-TEM, and pEscP_1-50_-TEM, expressed fusion proteins of the full-length EscP and EscP N-terminal fragments and TEM-1.

To clone the *escP* gene into the overexpressing vector, the coding sequence of *escP* was amplified by PCR with primers that fused a Flag tag to the coding region at the C terminus of the protein (Table 1). The PCR product was then subcloned as an NdeI/HindIII fragment into NdeI/HindIII-digested pET21a. To construct EscP-Flag fused to GST, the *escP*-Flag fragment was amplified by PCR from the pEscP-Flag pET21 plasmid (Table 1). The PCR product was subcloned into a BamHI/XhoI-digested pGEX-6P-1 vector. The resulting plasmid expressed N-terminal glutathione *S*-transferase fused to the EscP-Flag protein. To construct expression vectors for SepL-His and SepD-His, the coding sequences of SepL and SepD were amplified by PCR from EPEC E2348/69 genomic DNA and subcloned as NcoI/XhoI fragments into NcoI/XhoI-digested pet-28a or pet-21a, respectively (Novagen). The *sepD* gene was also amplified with primers F_SepD_V5/R1_SepD_V5/R2_SepD_V5 (Table 1) to fuse a V5 tag to its C terminus. The PCR product was digested with BamHI/SalI and ligated into a BamHI/SalI-digested pACYC184 vector. All constructs were verified by DNA sequencing and are listed in Table 2. The EPEC strains containing a Flag fusion to chromosomal *sepD* or *sepL* was a generous gift from José L. Puente.

**TABLE 2:** Strains and plasmids used in this study

Strain or plasmid	Description	Reference
Strains		
WT EPEC	EPEC strain E2348/69, streptomycin resistant	62
EPEC Δ*escP*	Nonpolar deletion of *escP*	This study
EPEC Δ*lee*	LEE pathogenicity island deletion mutant	This study
EPEC Δ*escF*	Nonpolar deletion of *escF*	63
EPEC Δ*escN*	Nonpolar deletion of *escN*	64
EPEC Δ*sepL*	Nonpolar deletion of *sepL*	34
EPEC Δ*sepD*	Nonpolar deletion of *sepD*	34
EPEC Δ*escP* Δ*escU*	*escP escU* double mutant	This study
Plasmids		
pEscP-Flag (pACYC)	C-terminally Flag-tagged EscP in pACYC184	This study
pEscP-V5 (pACYC)	C-terminally V5-tagged EscP in pACYC184	This study
pEscP-Flag (pET21a)	C-terminally Flag-tagged EscP in pET21(+)	This study
pEscP-2HA	C-terminally tagged EscP in pTOPO-2HA	This study
pEscI-V5	C-terminally V5-tagged EscI in pACYC184	18
pCX341	β-Lactamase	49
pEspZ-TEM	EspZ fused to β-lactamase	49
pEscP-TEM	EscP fused to β-lactamase	This study
pEscP_1-20_-TEM	20 N-terminal amino acids of EscP fused to β-lactamase	This study
pEscP_1-50_-TEM	50 N-terminal amino acids of EscP fused to β-lactamase	This study
pSepL-2HA	C-terminally tagged SepL in pTOPO-2HA	34
pSepD-2HA	C-terminally tagged SepD in pTOPO-2HA	34
pSepL-His	C-terminally tagged SepL in pET28(+)	This study
pSepD-His	C-terminally tagged SepD in pET21(+)	This study
pEscP-Flag (pGEX)	C-terminally tagged EscP in pGEX	This study
pSepD-V5	C-terminally V5-tagged SepD in pACYC184	This study
pCR2.1-TOPO	PCR cloning vector, Amp^r^ Kan^r^	Invitrogen
pET28a(+)	Expression vector for His tagging, Kan^r^	Novagen
pET21a(+)	Expression vector, Amp^r^	Novagen
pTOPO-2HA	Expression vector for HA tagging, Kan^r^	40
pACYC184	Cloning vector, Cm^r^ Tc^r^	65
pRE112	Suicide vector for allelic exchange, Cm^r^	55

### Immunoblotting.

Samples were subjected to SDS-PAGE and transferred to nitrocellulose membranes (Bio-Rad). Blots were blocked for 1 h in 5% (wt/vol) skim milk–PBST (0.1% Tween 20 in phosphate-buffered saline [PBS]) and then incubated with the primary antibody diluted in 3% skim milk–PBST for 1 h at room temperature. The optimal dilution of each antibody was calibrated individually. The secondary antibody was diluted in 3% skim milk–PBST, incubated with the blots for 1 h at room temperature, and detected with the ECL reagents (Biological Industries). The primary antibodies were mouse anti-V5 (Invitrogen), mouse anti-FLAG (Sigma), rabbit anti-maltose-binding protein (anti-MBP; Thermo Fisher Scientific), mouse anti-His (Thermo Fisher Scientific), mouse anti-DnaK (Abcam, Inc.), mouse anti-β-lactamase (Abcam, Inc.), mouse anti-HA (Abcam, Inc.), rabbit anti-GST (a gift from Noah Isakov), rat anti-HA (Roche), rabbit anti-ATPase β subunit, and rabbit anti-OmpA (a gift from Francisco Garcia-Del Portillo) antibodies. Antibodies directed against T3SS components were generated in the Finlay laboratory and included rabbit anti-EscF, mouse anti-EspB, rat anti-EscJ, mouse anti-Tir, and rat anti intimin antibodies. The secondary antibodies were horseradish peroxidase (HRP)-conjugated goat anti-mouse (Abcam, Inc.), HRP-conjugated goat anti-rabbit (Abcam, Inc.), and HRP-conjugated goat anti-rat (Jackson ImmunoResearch) antibodies.

### Secretion assay.

EPEC strains were grown overnight at 37°C in LB broth with appropriate antibiotics. The cultures were diluted 1:50 in DMEM (preheated overnight) and grown in a tissue culture incubator (with 5% CO_2_) statically for 6 h, and their OD_600_ was measured. The cultures were centrifuged at 20,000 × *g* for 5 min to remove the bacteria, the bacterial pellets were dissolved in SDS-PAGE sample buffer, and the supernatants were collected and then filtered through a 0.22-μm filter (Millipore). The supernatants were then precipitated with 10% (vol/vol) trichloroacetic acid overnight at 4°C to concentrate proteins secreted into the culture medium. The volume of the supernatants was normalized to the OD_600_ of the bacterial cultures to ensure equal loading of the samples. The samples were then centrifuged at 20,000 × *g* for 45 min at 4°C. The secreted protein precipitates were dissolved in SDS-PAGE sample buffer, and the residual trichloroacetic acid was neutralized with saturated Tris. The proteins were analyzed by SDS–12% PAGE and stained with InstantBlue (Expedeon).

### Calcium spike.

Regular DMEM (1.8 mM CaCl_2_, simulating the extracellular calcium concentration) and low-calcium DMEM (120 nM CaCl_2_, simulating the intracellular calcium concentration) were preheated overnight in a CO_2_ tissue culture incubator. WT EPEC expressing the EspZ-TEM fusion protein was grown overnight in LB broth at 37°C. The bacterial culture was washed once with modified PBS (without CaCl_2_ and MgCl_2_) to remove residual LB broth and resuspended in 1 ml of modified PBS. The washed culture was diluted 1:50 in preheated regular and low-calcium DMEM. The bacteria were grown statically in a tissue culture incubator (with 5% CO_2_) for 2.5 h, and then their OD was measured. Normalized according to their OD_600_, bacteria were taken from their original growth medium; washed twice with modified PBS; resuspended in fresh, preheated regular or low-calcium DMEM; and grown for an additional 45 min. The OD of the samples was measured again, and normalized volumes of supernatants were collected and concentrated as described above. The samples were subjected to SDS-PAGE and then analyzed by immunoblotting.

### Translocation assay.

Translocation of TEM-1 fusions into HeLa cells was carried out as previously described (45, 57), with slight modifications. Briefly, WT and Δ*escN*, Δ*escP*, and Δ*sepL* mutant EPEC strains expressing the EspZ–TEM-1 fusion protein were grown overnight at 37°C in LB broth, washed in modified PBS (without calcium and magnesium) to remove residual LB broth, and preinduced in regular or low-calcium DMEM for 2.5 h to an OD_600_ of 0.1 before being used to infect HeLa cells (3 × 10^4^ cells/well) at a multiplicity of infection of 1:100. Bacterial infection of HeLa cells was done in either regular or low-calcium DMEM. Thirty minutes after the addition of the bacteria to HeLa cells, protein expression was induced with 1 mM isopropyl-β-d-thiogalactopyranoside (IPTG) for an additional hour. Cell monolayers were then washed twice with PBS and incubated for 1 h at room temperature with freshly prepared CCF2/AM (Invitrogen). The plate was excited at 405 nm, and emissions were recorded at 460 and 530 nm (SpectraMax Paradigm; Molecular Devices). Relative TEM-1 translocation efficiency was expressed as the emission ratio of the cleaved CCF2 (blue; 460 nm) and the original CCF2 (green; 530 nm) in accordance with the manufacturer’s instructions.

### Bacterial cell fractionation.

Bacterial cell fractionation was based on previously described procedures (58). Briefly, EPEC (WT and Δ*lee* mutant) strains from an overnight culture were subcultured 1:50 in 50 ml of DMEM for 6 h at 37°C in a CO_2_ tissue culture incubator. The cultures were harvested, washed in PBS, and resuspended in 1 ml of buffer A (50 mM Tris [pH 7.5], 20% [wt/vol] sucrose, protease inhibitor cocktail [Roche Applied Science], and lysozyme [100 µg/ml]) and incubated for 30 min at room temperature to generate spheroplasts. MgCl_2_ was then added to a final concentration of 20 mM, and samples were spun for 10 min at 5,000 × *g*. The supernatants containing the periplasmic fractions were collected. The pellets, which contained the cytoplasm and membrane fractions, were resuspended in 1 ml of lysis buffer (20 mM Tris-HCl [pH 7.5], 150 mM NaCl, 3 mM MgCl_2_, 1 mM CaCl_2_, and 2 mM β-mercaptoethanol with protease inhibitors). All subsequent steps were carried out at 4°C. Ten micrograms of RNase A and DNase I per milliliter was added, and the samples were sonicated (Fisher Scientific, 3 × 15 s). Intact bacteria were removed by centrifugation at 2,300 × *g* for 15 min, and the cleared supernatants containing cytoplasmic and membrane proteins were transferred to new tubes. To obtain the cytoplasmic fraction, supernatants were centrifuged in a Beckman TLA 100 Ultracentrifuge with a TLA100.3 rotor for 30 min at 100,000 × *g* to pellet the membranes. The supernatants containing the cytoplasmic fraction were collected. To separate the membrane fraction into IM and OM fractions, the washed membrane pellets were first resuspended in 0.1 ml of lysis buffer with 0.5% *N*-lauroylsarcosine, which selectively solubilizes the IM, and then centrifuged at 100,000 × *g* for 1 h. The supernatants, which contained the IM fraction, were collected, and the OM pellets were washed in lysis buffer with 0.5% *N*-lauroylsarcosine. The final pellets were resuspended in 0.1 ml of lysis buffer with 0.5% *N*-lauroylsarcosine and 0.1% SDS. The protein contents of all samples were determined by the Coomassie Plus protein assay (Thermo Fisher Scientific) before the addition of SDS-PAGE sample buffer with β-mercaptoethanol. Membrane fractionation with a sucrose density gradient was based on previously described protocols (59–61). An Δ*escP* mutant EPEC strain containing either EscP-Flag or EscP-V5 (pACYC) was subcultured from an overnight culture 1:50 in 500 ml of DMEM for 6 h at 37°C in a CO_2_ tissue culture incubator. The cultures were harvested, washed in PBS, and resuspended in 8 ml of buffer A (50 mM triethanolamine [pH 7.5], 250 mM sucrose, 1 mM EDTA, 1 mM dithiothreitol [DTT], and protease inhibitor cocktail [Roche]). The cells were lysed by sonication (Fisher Scientific; 2 × [3 × 5 s], amplitude 30%) and centrifuged for 20 min at 8,000 × *g* to remove intact bacteria. All subsequent steps were carried out at 4°C. Six milliliters of the supernatant was overlaid on top of a two-step sucrose gradient comprising 1 ml of 55% (wt/wt) sucrose on the bottom and 5 ml of 9% (wt/wt) sucrose on the top. The gradient was spun in a Beckman SW-41 rotor for 2 h at 240,000 × *g*. One milliliter of crude membrane fraction was collected from the top of the 55% step, diluted 1:1 in buffer B (50 mM triethanolamine [pH 7.5], 1 mM EDTA, 1 mM DTT, and protease inhibitor cocktail), and then subjected to further fractionation on a six-step sucrose gradient (from bottom to top, 1 ml of 55%, 2 ml of 50%, 2 ml of 45%, 2 ml of 40%, 2 ml of 35%, and 1 ml of 30% sucrose) and spun for 14 h at 210,000 × *g*. All sucrose gradients were prepared in buffer B. The top 2 ml of the spun gradient was discarded, and 1-ml fractions were collected from top to bottom, diluted 1:1 in buffer B, and centrifuged for 1 h at 210,000 × *g*. The pellets were resuspended in 100 µl of sample buffer and analyzed by Western blotting.

### Purification of SepL-His, SepD-His, and EscP-Flag.

*E. coli* strain BL21 transformed with pGST-EscP-Flag was grown to mid-exponential phase in LB broth and induced with 0.1 mM IPTG for 16 h at 18°C. The bacteria were harvested and resuspended in PBS buffer containing 0.5 mg/ml lysozyme and incubated for 30 min on ice. After the subsequent addition of a protease inhibitor cocktail and sonication (Fisher Scientific, 3 × 15 s), the bacterial lysate was centrifuged, collected, mixed with glutathione-Sepharose 4B beads (GE Healthcare), and incubated for 1.5 h at 4°C on a rotatory wheel. The beads were then washed, and the bound protein was subjected to cleavage from GST by overnight incubation at 4°C with PreScission protease (GE Healthcare). The cleaved EscP-Flag protein was further purified by gel filtration chromatography with a Superdex 200 HR 10/300 column (GE Healthcare) calibrated to a mixture of 20 mM Tris (pH 8.0), 150 mM NaCl, and 5% glycerol. The peak fractions were concentrated, and the protein was frozen in liquid nitrogen and stored at −80°C. The GST protein that was used as a negative control for some experiments was overexpressed, purified by a procedure similar to that described above, and then eluted by competition with l-glutathione (Sigma). To purify SepL-His and SepD-His, strains of *E. coli* BL21 transformed with pSepL-His (pET28) or pSepD-His (pET21) were grown to mid-exponential phase and induced for 4 h at 26°C in the presence of 1 mM IPTG. The His-tagged proteins were purified with Ni-nitrilotriacetic acid resin (Thermo Fisher Scientific) according to the manufacturer’s protocol. For further purification, SepD-His was loaded onto a HiTrap Q HP column (GE Healthcare), followed by a Superdex 200 HR 10/300 column, and SepL-His was loaded onto a Superdex 200 HR 10/300 column under conditions similar to those described above.

### Co-IP assays.

EPEC strains transformed with pEscP-Flag, pSepL-2HA, or both were subcultured 1:40 into 50 ml of preheated DMEM and grown at 37°C as a static culture with 5% CO_2_ for 6 h. The cells were harvested by centrifugation at 4,000 × *g* for 15 min at 4°C and washed twice with PBS. The washed pellets were resuspended in lysis buffer containing 20 mM Tris-HCl (pH 7.4), 120 mM NaCl, 2 mM MgCl_2_, 1 mM CaCl_2_, 2.5% glycerol, 0.5 mM β-mercaptoethanol, 0.1% NP-40, and protease inhibitor cocktail. For experiments designed to detect calcium-dependent protein interactions, the lysis buffer was prepared without CaCl_2_ and included ethylene-diamine-tetraacetic acid (EDTA) (Calbiochem) or BAPTA (Millipore), as indicated in Results. Following sonication, intact cells were removed by centrifugation at 18,000 × *g* at 4°C for 10 min. To reduce nonspecific binding, the lysates were preincubated with 60 µl of washed protein G slurry beads (Millipore) for 30 min at 4°C on a rotatory wheel. The precleared lysate was collected and incubated with 1.5 µg of mouse anti-HA antibody for 30 min at 4°C, and then 30 µl of washed protein G slurry beads was added to each sample, which was incubated on a rotatory wheel for 2 h at 4°C. Finally, the beads were centrifuged, washed five times with 1 ml lysis buffer, and eluted by adding 50 µl of SDS-PAGE sample buffer and boiling the beads for 5 min. Equal amounts of WCL and eluted fractions were subjected to SDS-PAGE and then Western blot analysis. To examine protein interaction *in vitro*, *E. coli* BL21 transformed with SepL-His, SepD-Flag, or EscP-Flag was grown to mid-exponential phase and induced with 1 mM IPTG for 4 h at 26°C. Co-IP experiments were done by a protocol similar to that described above, except that 1.5 µg of anti-Flag antibody was added to each sample and after binding of the Flag-labeled proteins to the beads, SepL-His lysates were added. The negative control was performed in the absence of anti-Flag antibody.

To examine whether the protein interactions are calcium dependent *in vitro*, a similar experimental setup was used, with a few modifications. The EscP-Flag-bound beads were washed five times with modified PBS (no calcium or magnesium), samples of *E. coli* BL21 expressing SepL-His were washed with modified PBS and resuspended under four conditions (Ca^2+^, no Ca^2+^, no Ca^2+^ with EDTA, and no Ca^2+^ with BAPTA) before sonication and WCL preparation. Similar amounts of EscP-Flag-bound beads were incubated with the SepL-His lysates prepared with different buffers for 2 h at 4°C on a rotatory wheel, washed five times, eluted, and subjected to SDS-PAGE and then Western blot analysis as described in the previous paragraph.

To examine whether EscP and SepD compete for interaction with SepL, lysates of *E. coli* BL21 expressing EscP-Flag were incubated with anti-Flag antibody and protein G slurry beads under conditions similar to those described above. EscP-Flag bound beads were divided equally into five samples, to each of which purified SepL-His and/or SepD-His were added in various amounts (described in Results). In addition, 2 mg/ml bovine serum albumin (BSA) was added to all samples to reduce nonspecific binding. The negative control was set up by a procedure similar to that used for the other samples but without the addition of anti-Flag antibody. The mixtures were incubated for 2 h at 4°C on a rotatory wheel, washed, and eluted, and samples were subjected to SDS-PAGE as described previously.

### ELISAs.

Ninety-six-well plates (Corning Costar) were coated overnight with 70 µl of 10 µg/ml purified SepL-His or 1% fetal calf serum (FCS; served as a negative control) in 1 M Na_2_HPO_4_ buffer (pH 9.0) at 4°C. The wells were then blocked for 1 h at 37°C with 300 µl of 10% FCS per well in binding buffer (Tris 20 mM [pH 7.4], 120 mM NaCl, 2 mM MgCl_2_, 1 mM CaCl_2_, 2.5% glycerol, and protease inhibitor cocktail [Roche]) and washed three times with binding buffer. For the EscP-SepD competition experiments, purified EscP-Flag and SepD-His were diluted to 15, 30, and 60 nM in binding buffer containing 2 mg/ml BSA to prevent unspecific interaction. For calcium sensitivity assays, EscP was prepared in binding buffer containing either CaCl_2_ or 2 mM BAPTA. Protein samples were incubated for 1 h at 37°C. The wells were then washed with Tris-buffered saline containing 0.1% Tween 20 (TBS-T) containing 1 mM CaCl_2_, blocked for 20 min with 10% FCS, and incubated with mouse anti-Flag antibody for 1 h at room temperature (RT). The wells were washed three times in TBS-T and incubated with goat anti-mouse antibody labeled with HRP for 40 min. Finally, the wells were washed seven times with TBS-T, incubated with 60 µl of TMB-Plus substrate solution (Dako) for 20 min at RT, and analyzed by spectrophotometry at 650 nm.
